# A Human PrM Antibody That Recognizes a Novel Cryptic Epitope on Dengue E Glycoprotein

**DOI:** 10.1371/journal.pone.0033451

**Published:** 2012-04-03

**Authors:** Annie Hoi Yi Chan, Hwee Cheng Tan, Angelia Yee Chow, Angeline Pei Chiew Lim, Shee Mei Lok, Nicole J. Moreland, Subhash G. Vasudevan, Paul A. MacAry, Eng Eong Ooi, Brendon J. Hanson

**Affiliations:** 1 Bio-Defence Programme, DMERI, DSO National Laboratories, Singapore, Singapore; 2 Department of Microbiology, National University of Singapore, Singapore; 3 Emerging Infectious Diseases Programme, Duke-NUS Graduate Medical School, Singapore, Singapore; 4 Center for BioImaging Sciences, National University of Singapore, Singapore; University of Pittsburgh, United States America

## Abstract

Dengue virus (DENV) is a major mosquito-borne pathogen infecting up to 100 million people each year; so far no effective treatment or vaccines are available. Recently, highly cross-reactive and infection-enhancing pre-membrane (prM)-specific antibodies were found to dominate the anti-DENV immune response in humans, raising concern over vaccine candidates that contain native dengue prM sequences. In this study, we have isolated a broadly cross-reactive prM-specific antibody, D29, during a screen with a non-immunized human Fab-phage library against the four serotypes of DENV. The antibody is capable of restoring the infectivity of virtually non-infectious immature DENV (imDENV) in FcγR-bearing K562 cells. Remarkably, D29 also cross-reacted with a cryptic epitope on the envelope (E) protein located to the DI/DII junction as evidenced by site-directed mutagenesis. This cryptic epitope, while inaccessible to antibody binding in a native virus particle, may become exposed if E is not properly folded. These findings suggest that generation of anti-prM antibodies that enhance DENV infection may not be completely avoided even with immunization strategies employing E protein alone or subunits of E proteins.

## Introduction

Dengue virus (DENV) is a flavivirus with four related but antigenically distinct serotypes (DENV1-4). It infects approximately 50–100 million people each year, of which 500,000 people exhibit the life-threatening form of severe dengue – dengue haemorrhagic fever (DHF) and dengue shock syndrome (DSS) [1]. The current lack of treatment or licensed vaccine means dengue poses a serious public health threat [2]. Infection by one serotype of DENV confers lifelong immunity against the homologous serotype, but only limited cross-protection to the remaining three serotypes [3], [4]. The presence of cross-reactive, non-neutralizing antibodies generated during a primary infection has been suggested to enhance the pathogenicity of subsequent infections via the process of antibody-dependent enhancement (ADE) [5]. A successful and safe vaccine candidate must therefore elicit a protective long-lasting immune response to all four serotypes [4], [6]–[8].

Recent immunological studies have shown the human anti-DENV immune response to be dominated by prM-specific antibodies in both primary and secondary infections [9], [10]. These prM-specific antibodies are highly cross-reactive and non-neutralizing. When complexed with immature DENV (imDENV), it has the ability to render normally non-infectious imDENV highly infectious [11], [12]. This has caused concern over current vaccine candidates that contain native dengue prM [9] – which is a component of most current vaccine strategies whether naturally attenuated, recombinantly attenuated, yellow fever-dengue-virus chimeras, chemically inactivated virus, DNA vaccine or recombinant subunit protein vaccines [13], [14]. Vaccine candidates that do not contain prM proteins, such as soluble recombinant Envelope (E) protein or E domain subunit vaccines may thus become increasingly important.

To gain a deeper understanding of the early DENV-specific immune response in humans, the four serotypes of DENV were sequentially screened with a non-immunized human Fab phage display library. Broadly cross-reactive prM-specific antibodies dominated the screen and the Fab with highest affinity, D29 Fab-IgG, was converted into full-length human IgG1 format for thorough characterization. This antibody (D29 Fab-IgG) was found to have high affinity for a conformational epitope on prM and, like other prM antibodies [11], [12], was capable of restoring the infectivity of virtually non-infectious immature DENV (imDENV) in FcγR-bearing K562 cells. The antibody also cross-reacted with E protein – fine mapping and site-directed mutagenesis studies localized the epitope to the DI/DII junction of E, which would be inaccessible in a native virus particle. This suggests the possibility that immunization strategies employing E protein alone or subunits of E proteins may not be able to completely avoid generation of anti-prM antibodies that enhance DENV infection.

## Materials and Methods

### Cells

*Aedes albopictus* C6/36 cells (ATCC) were maintained in Leibovitz L-15 media (GIBCO, Invitrogen) supplemented with 8% fetal bovine serum (FBS), at 28^o^C, 5% CO_2_. BHK-21 cells (ATCC) and human erythroleukemic K562 cells (ATCC) were maintained in RPMI 1640 GlutaMAX medium (RPMI) (GIBCO, Invitrogen) containing 10% FBS, and incubated at 37^o^C, 5% CO_2_. Vero cells (ATCC) were grown in 199 medium (M199) (GIBCO, Invitrogen) supplemented with 8% FBS, 1% sodium pyruvate and 1% NEAA. HEK293 T cells (ATCC) was cultured in Dulbecco’s modified Eagle’s medium (DMEM) (GIBCO, Invitrogen) supplemented with 10% FBS at 37^o^C in 5% CO_2_.

### Antibodies and Proteins

Mouse monoclonal antibodies 3H5 (m3H5), 4G2 (m4G2) and 2H2 (m2H2) are specific to EDIII of DENV2, EDII of flaviviruses and prM of DENV1-4 respectively. Chimeric humanized 3H5 (h3H5) and 4G2 (h4G2) were constructed by cloning the Fab portion (variable light and heavy chains) into an expression vector containing the human IgG1 framework for expression in HEK293 T cells [15]. Conjugation of HRP to antibodies-D29, -m3H5 and -m2H2, was performed using the Lightning-Link HRP Conjugation Kit (Innova Biosciences).

### Virus Growth

Dengue 1 (DENV1) strain Hawaii, Dengue 2 (DENV2) strains New Guinea C (NGC) and ST, Dengue 3 (DENV3) strain H87 and Dengue 4 (DENV4) strain H241 were passaged in Vero cells for all assays unless otherwise stated. Virus titers were determined by plaque assay on BHK cells. Immature DENV (imDENV) was produced as described previously [16]. Briefly, the media of infected Vero cells was replaced 2 dpi with M199 NH_4_Cl medium (M199 with 10% FBS, 1% sodium pyruvate, 1% non essential amino acids, 1% PS, 2.5 mM L-Glutathione and 20 mM NH_4_Cl). Culture supernatants were harvested after 5 days and imDENV was purified by PEG precipitation. Virus titer was determined as genome containing particles (GCP)/ml by quantifying real-time PCR (qPCR) described previously [17].

### Direct Binding ELISA

To ascertain the binding specificity of D29 Fab-IgG, direct binding ELISA against dengue virus was performed using standard ELISA methods. Briefly, 2×10^5^ pfu/well purified DENV1-4 was coated on Maxisorb plate followed by blocking using 5% SM. Following incubation with 1 µg/ml dengue-specific antibodies, plates were probed with HRP-conjugated anti-human IgG-Fc secondary antibody or anti-mouse IgG-Fc secondary antibody (Pierce). Detection steps in subsequent ELISAs followed this method unless otherwise stated. To determine the binding affinity of D29 Fab-IgG direct binding ELISA was performed with serially diluted D29 Fab-IgG. Results were fitted to a one-site binding hyperbola using Prism 5.03 (GraphPad, San Diego, USA).

For Peptide phage ELISA, 2 µg of D29 and non-related control antibodies were coated on Maxisorb plate and incubated with phage, followed by detection with HRP-conjugated anti-M13 secondary antibody.

### Indirect Immunofluorescent Assay (IFA)

DENV-infected Vero cells or C6/36 cells were fixed and permeablilized with 80% acetone at −20^o^C for 10 min. The presence of virus was detected by D29 Fab-IgG, h3H5 and h4G2 at 1 µg/ml, followed by staining with FITC-conjugated anti-human IgG (ZyMax, Invitrogen). Cells were mounted with MOWIOL (Sigma) and images were captured using a fluorescent microscope (Olympus).

### Preparation of Dengue lysate and Western Blot

DENV-infected Vero cells were harvested 5 days post-infection and lysed with cold lysis buffer (1.5% Triton X-100, 0.2 µM Phenylmethylsulfonyl Fluoride (PMSF), 1 µg/ml pepstatin A and 75 µM KCl in PBS) for 40 min on ice. An equal volume of 5× loading dye was added to the cell lysate before 12% SDS-PAGE and transferred onto nitrocellulose membranes. Blocked membranes were probed with dengue-specific antibodies at RT for 1 hr and detected with HRP-conjugated anti-human IgG-Fc or anti-mouse IgG-Fc secondary antibody and developed with chemiluminescence (Supersignal West Pico Chemiluminescent Substrate, Pierce).

### Competition ELISA and Western Blot

To establish the binding target of D29 Fab-IgG, competition ELISA against epitope-characterized monoclonal antibodies was carried out. Plates were prepared as for direct binding ELISA. After blocking, serially diluted m3H5, m4G2 or m2H2 was added to the plates and incubated for 1 hr at RT, followed by addition of D29 Fab-IgG at 2.5 µg/ml and incubation for 1 hr at RT. Bound antigen-antibody complexes were detected by HRP-conjugated anti-human IgG-Fc secondary antibody (Pierce), and developed as described above.

For competition Western, Fab-IgG D29 was incubated with m3H5, m4G2 or m2H2 for 1 hr at RT before applying to blocked membranes for 30 min at RT. HRP-conjugated anti-Human IgG-Fc secondary antibody was applied to detect bound D29 for 45 min at RT after 4 washes with PBST.

### Immunoprecipitation

For radioactive immunoprecipitation, DENV2- infected BHK-21 cells were harvested 48 hr later and prepared as described previously [18]. For immunoprecipitation with SDS to disrupt protein complexes, lysate from infected cells was incubated with 1.25% SDS for 30 min at 4^o^C. The SDS concentration was diluted to 0.2% before immunoprecipitation and precipitated proteins were analysed by silver stained SDS-PAGE (Silver stain Plus Kit, Bio-Rad) and Western blot.

### Phage-displayed Random Peptide Library

The phage-displayed-12 random dodecapeptide (Ph.D-12) library (New England Biolabs) was panned against D29-Fab-IgG to identify the antibody epitope as per instruction manual. The first round of panning was carried out with 100 µg/ml D29 Fab-IgG immobilized on Maxisorb Immunotube (Nunc). The amplified phage was enriched by three further rounds of panning in solution using Protein A or G sepharose in alternate rounds of panning. One round of negative selection with Protein A and G sepharose was carried out to minimize non-specific binders. The isolated peptide phage sequences were mapped against 3D protein structure using the Peptiope server with crystal structures of prM-E heterodimer at neutral pH (Protein Data Bank (PDB) accession code 3C6E). Graphic visualization was carried out using MSI WebLab ViewLite (Accelrys).

To test whether isolated peptide phage inhibits binding of D29 to its natural epitope, inhibition ELISA was carried out. 10-fold serially diluted peptide phage (starting at 10^12^ pfu/ml) or 2×10^5^ pfu DENV2 was incubated with D29 Fab-IgG for 1 hr before applying to DENV2-coated plate for 5 min at RT. Bound antibody was detected with HRP-conjugated anti-human IgG-Fc secondary antibody for 1 hr at RT.

To test the inhibition capacity of peptide phage by Western blot, pre-incubation of 0.1 µg/ml of D29 Fab-IgG with 4×10^11^ pfu peptide phage; 8×10^6^ pfu DENV2 or 5%SM for 1 hr was carried out before applying to membrane transblotted with DENV2 lysate for 30 min. HRP-conjugated anti-Human IgG-Fc secondary antibody was applied to detect bound D29 Fab-IgG for 45 min at RT and the membrane was processed as described previously to visualize the reactive bands.

**Figure 1:**
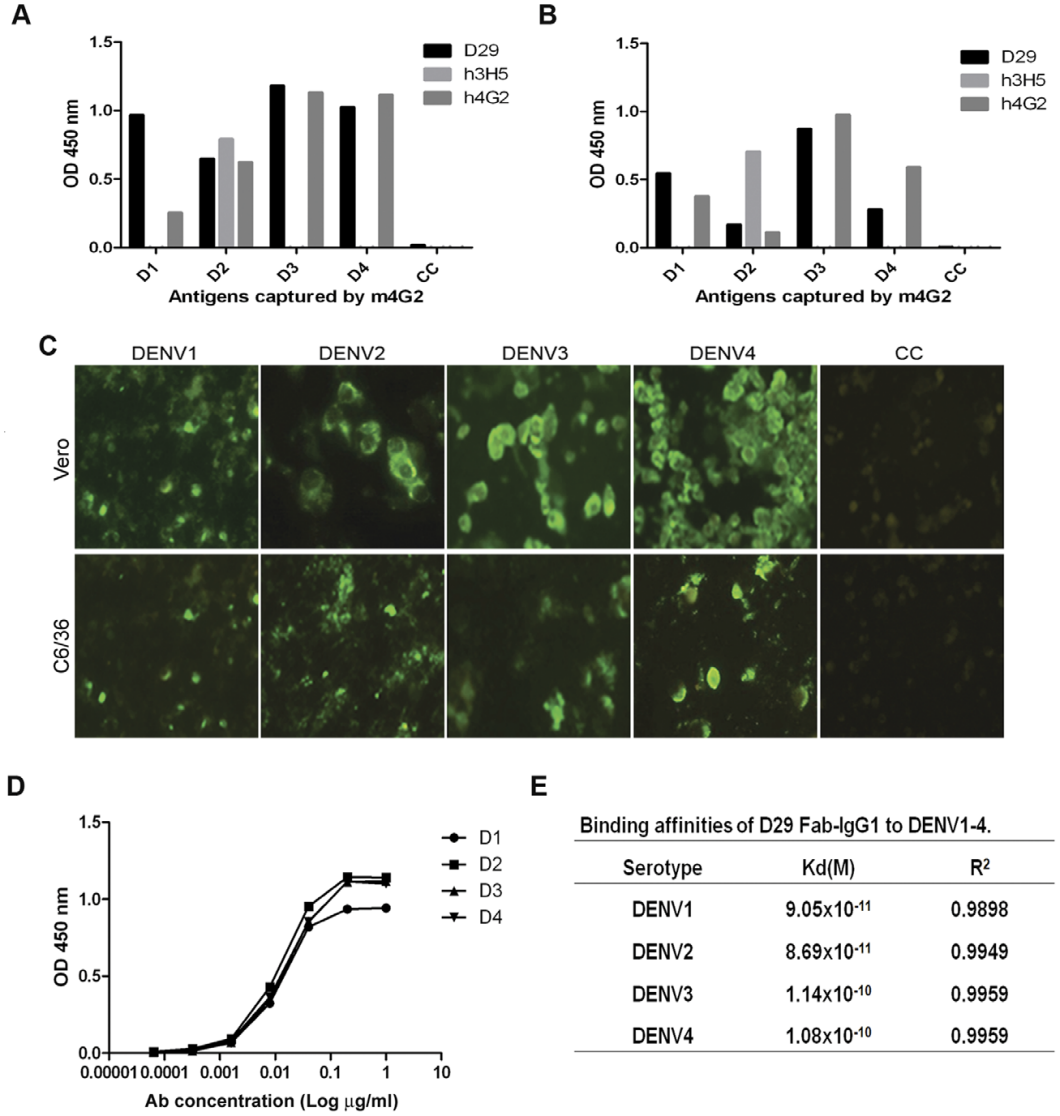
Binding specificity and affinity of D29 Fab-IgG. To determine the binding specificity of D29 Fab-IgG in a sandwich ELISA format, DENV2 in infected (A) Vero or (B) C6/36 cell culture media was captured by immobilized m4G2 and detected by 1 µg/ml D29 Fab-IgG, h3H5 and h4G2. (C) Immunoreactivity of D29 Fab-IgG was also tested in the IFA format against DENV1-4 infected Vero (Ve) or C6/36 (C6) cells. Binding of D29 Fab-IgG was detected by anti-human-IgG-FITC. Non-infected cells (CC) were included as control. (D) To determine its binding affinity, DENV1-4 was detected by serially-diluted D29 Fab-IgG in a direct binding ELISA format. Values displayed are the average of three independent experiments with error bars representing the standard errors of the mean. Results were fitted to a one-site binding hyperbola using GraphPad Prism 5.03 (GraphPad, San Diego, USA) to determine the Kd values for each DENV serotype. (E) Summary of the Kd and R^2^ values of D29 Fab-IgG for each DENV serotype.

### Site-directed Mutagenesis

The gene portion containing prM-E, as described in *Puttikhunt, et al* [24], was amplified from DENV2 (NGC) cDNA using the primers D2-FW (5′-AATTAATACGACCGTCTCCCATGAATAGAAGACGCAGATCTGCAGGC-3′) and D2-RV (5′-CGCCCGTTTGATCTCGAGCTACTAGGCCTGCACCATGACTCCC-3′) and cloned into pCMV-myc-ER vector via the restriction sites Nco I and Xho I to create plasmid construct pCMV-prM-E. Using this as template, selected amino acids residues of the predicted epitopes, P3 and P9, were mutated by site-directed mutagenesis using Quikchange Multi Site-directed Mutagenesis kit (Stratagene, Agilent Technologies). PCR reactions and subsequent cloning steps were carried out according to manufacturer’s instructions. Substitution of amino acids in all mutant constructs was confirmed by sequencing. Mutants were expressed in HEK293-T cells and harvested 48 hr post-transfection for immunoblot analysis and immunoprecipitation experiments.

### Plaque Reduction Neutralization Test (PRNT) and ADE Assay

To determine the ability of D29 Fab-IgG to neutralize Dengue infection *in vitro*, PRNT was carried out on BHK cells as described previously [19].

The ADE assay was performed as described previously [20]. Briefly, pre-formed immune complexes were prepared by incubating serially diluted antibody with DENV2 or imDENV2 at a multiplicity of 25 GCP per cell (MOG 25) in RPMI MM at 37^o^C for 1 hr before applying to 2×10^4^ K562 cells in 96-well U-bottom plate (Nunc). Supernatant was harvested 48 hr post-infection and virus titer was quantified by plaque assay.

## Results

### Binding Specificity and Affinity of D29 Fab-IgG

Following isolation from the human non-immune Fab-phage library by panning against the 4 serotypes of DENV sequentially (Fig. S1), D29 Fab-IgG was converted into full length human IgG1 (Materials and Methods S1). To ensure the antibody was specific for DENV viral proteins, ELISA and IFA were performed with all 4 serotypes of DENV grown in C6/36 and Vero cell lines. D29 Fab-IgG reacted with infected cells from DENV1-4 without cross-reaction to the non-infected cell controls, indicating that the determinant for D29 Fab binding is indeed viral and not host (Fig. 1A, B, C).

Using direct ELISA, D29 Fab-IgG was found to have high affinity for all 4 serotypes of DENV (Fig. 1D and E): DENV2(8.69×10^−11^ M)∼DENV1(9.05×10^−11^ M)>DENV4(1.08×10^−10^ M)∼DENV3(1.14×10^−10^ M). However, the amino acid sequence of its CDRs was found to have little deviation from germline (Fig. S2), indicating D29 has not been affinity matured via somatic hypermutation. This suggests that antibodies like D29 can be generated in the early immune response against DENV.

**Figure 2:**
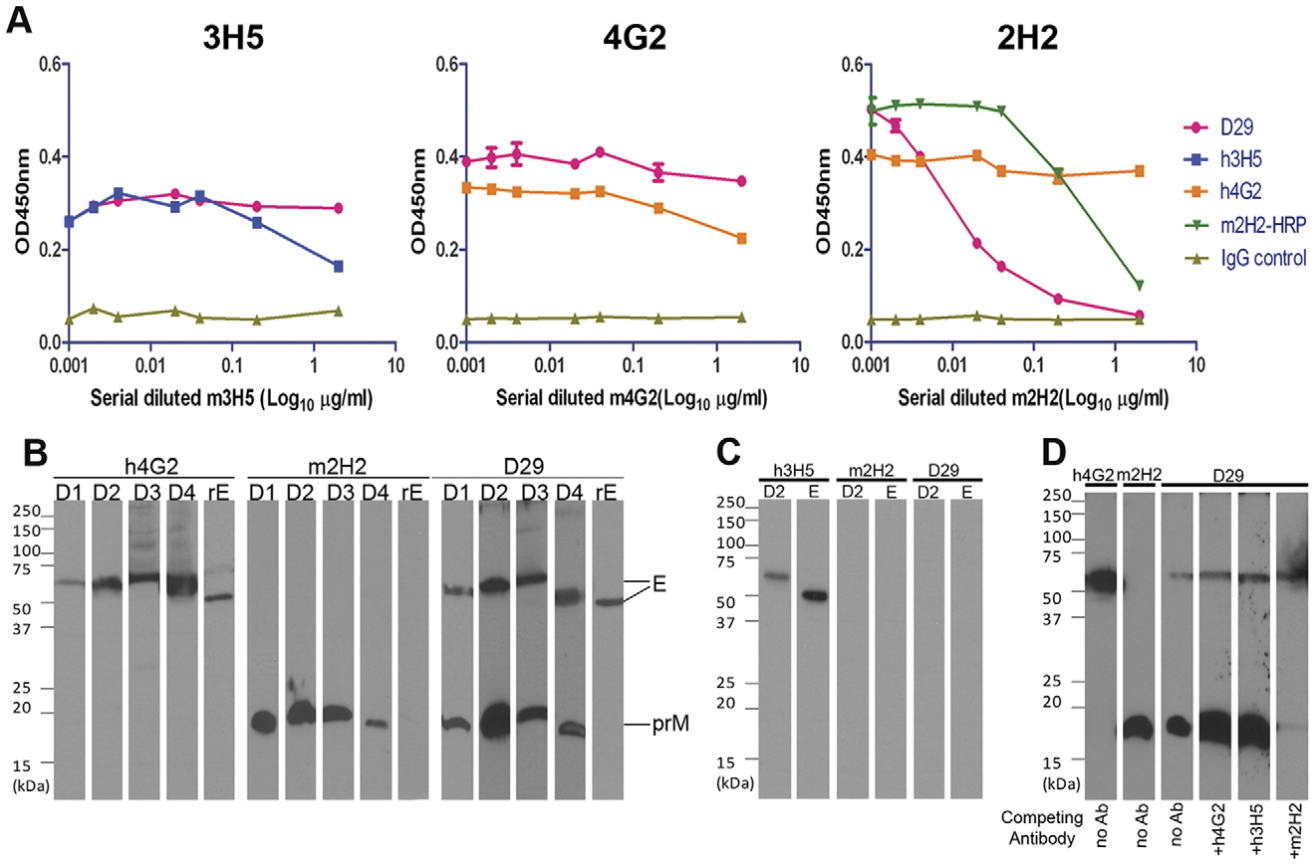
Competition Assays and Western Blots of D29 Fab-IgG and monoclonal anti-DENV antibodies with known epitope. (A) Serially diluted h3H5, h4G2 or m2H2 was incubated with immobilized DENV2 (2×10^7^ pfu/ml) for 1 hr at RT before addition of 2.5 µg/ml of D29 Fab-IgG for a further hour at RT. Bound D29 Fab-IgG was detected by HRP-conjugated anti-human IgG-Fc antibody. 2.5 µg/ml of HRP-conjugated m2H2 was used to compete against m2H2. Values displayed are the average of three independent experiments and error bars represent standard errors of the mean. For Western Blot analysis, DENV1-4 (D1-D4) infected Vero cell lysate and 0.5 µg of recombinant DENV2 E protein (ecto-domain) (rE) were separated on 12% SDS-PAGE in (B) non-reducing, (C) reducing conditions; followed by detection with 1 µg/ml of h3H5, h4G2, m2H2 and D29 Fab-IgG. (D) For competition Western blot analysis, 0.5 µg/ml of D29 Fab-IgG was incubated with 1 µg/ml of h4G2, h3H5, m2H2 for 1 hr at RT before applying to membrane transblotted with DENV2 viral lysate for 30 min at RT.

### Identification of the Binding Target of D29-Fab IgG

To broadly characterize the epitope of D29 Fab-IgG on DENV, we used competition ELISA with well characterized DENV antibodies including mouse-derived 3H5 (m3H5) which is specific to DENV2 EDIII; m4G2, a cross-reactive antibody that recognizes EDII of all flaviviruses and m2H2, a cross-DENV reactive anti-prM antibody [21], [22]. Making use of humanized versus parental murine antibodies for 3H5 and 4G2, and HRP-conjugated m2H2 versus non-conjugated for m2H2, each of the reference antibodies was shown to self-compete (Fig. 2A). No significant competition was observed between D29 Fab-IgG and m3H5, m4G2 or non-DENV specific control antibody. However, binding of D29 Fab-IgG to DENV2 decreased significantly in the presence of m2H2 in a dose-dependent manner, indicating that D29 Fab-IgG recognized an epitope on prM similar to that of m2H2 (Fig. 2A).

To confirm the target protein of D29 Fab-IgG, Western blot analysis was carried out with DENV-infected Vero cell lysates and the recombinant ectodomain of DENV2 E protein (rE) under reducing and non-reducing conditions. Under non-reducing conditions, the control antibodies recognized their respective target proteins from the cell lysates: h4G2 detected the ∼55 kDa E protein of all 4 serotypes and the ∼50 kDa rE protein, and m2H2 detected the ∼20 kDa prM of all 4 serotypes (Fig. 2B). Remarkably, D29 Fab-IgG detected both E and prM of all 4 serotypes; however, the binding was abolished in the presence of reducing agent, indicating that the D29 epitope is conformational. All control antibodies except h3H5 lost binding to their target proteins under reducing condition (Fig. 2C), which is expected since h3H5 recognizes a linear epitope on EDIII [22], [23], whereas both h4G2 and m2H2 are conformationally sensitive [10], [24]. In agreement with the competition ELISA data, h3H5 and h4G2 did not compete with D29 Fab-IgG for epitopes in the Western blot format, whereas incubation with m2H2 abolished D29 binding to prM but not to E protein (Fig. 2D).

**Figure 3:**
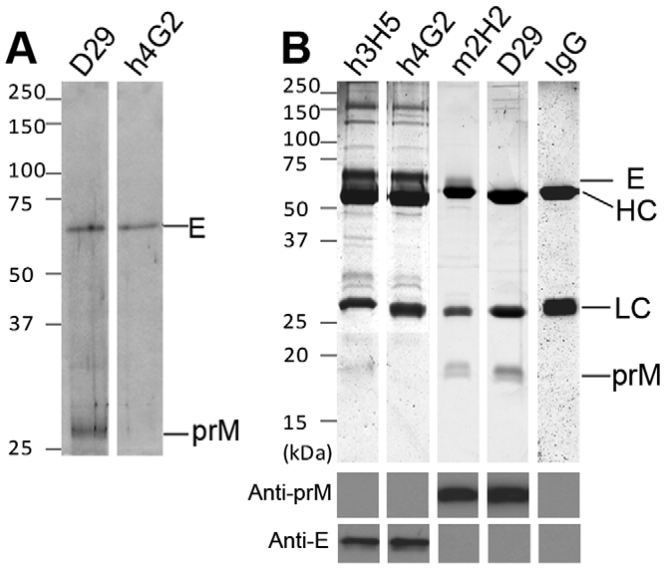
Immunoprecipitation of DENV2 proteins with anti-DENV antibodies. (A) For immunoprecipitation in native condition, DENV2-infected BHK cells were incubated with media supplemented with 250 µCi/ml ^35^S-methionine before lysis. Viral proteins were precipitated with D29 Fab-IgG or h4G2. (B) For immunoprecipitation with 1.25% SDS, cleared lysate of DENV2-infected BHK cells were incubated with h3H5, h4G2, m2H2, D29 Fab-IgG or non-DENV specific human antibody (IgG) followed by analysis with silver staining and Western blotting. Precipitated prM and E proteins were verified by (anti-prM) HRP-conjugated m2H2 or (anti-E) h3H5 respectively. HC - antibody heavy chain; LC - antibody light chain.

Given the ability of D29 Fab-IgG to bind both prM and E on western blots, immunoprecipitation was carried out with DENV2-infected BHK cell lysate to elucidate if D29 Fab-IgG binds both proteins in solution. An initial radioactive immunoprecipitation showed D29 Fab-IgG binding to both E (∼55 kDa) and prM (∼20 kDa), as both proteins are precipitated whereas h4G2 only precipitated E protein (Fig. 3A). However, as prM intrinsically interacts with E to form heterodimers, it is possible that antibody binding to one of the components in the heterodimer would pull down the other. To address this, dissociation of the heterodimer with 1.25% SDS was performed prior to the assay. Immunoprecipitated proteins were resolved by reducing SDS-PAGE and visualized by silver staining. Identity of the precipitated proteins was verified by non-reducing Western blot analysis with h3H5 and m2H2, respectively (Fig. 3B). The control antibodies were able to precipitate their corresponding binding partners. Despite the ability to recognize both prM and E on western blots, D29 Fab-IgG only precipitated prM. Taken collectively, our data indicate that D29 binds both prM and E but the epitope on E is not accessible on the native protein.

### Fine Epitope Mapping by Phage-displayed Random Peptide Library Screening

As the DTT-sensitive m2H2 had previously been mapped to its epitope using linear peptides [21], we attempted a similar approach to locate the binding epitope of D29 Fab-IgG. An inhibition ELISA was carried out using synthetic ∼20 mer linear peptides covering DENV2 prM [25] as well as a library of short 15-mer overlapping linear peptides (Mimotope) corresponding to aa1–166 of prM and aa1–495 of E (Materials and Methods S1). However, none of the linear peptides were able to significantly inhibit the binding of D29 Fab-IgG to DENV2 (Fig. S3), suggesting that the epitope of D29 Fab-IgG1 is discontinuous.

Next, D29 Fab-IgG was screened using a random dodecapeptide (Ph.D-12)-phage library as this approach has previously been used to map antibody-binding motifs [26], [27]. Peptide-phage clones were randomly selected from the second, third and fourth round of panning, and their binding specificity was confirmed by direct binding ELISA against D29 Fab-IgG. Reactive clones were sequenced and checked against a list of common target-unrelated peptides (TUPs) [28] to eliminate peptides that react with constant regions of antibodies, protein A/G sepharose or plastic surfaces. No TUPs were detected and the resulting 20 unique clones fall into three main consensus groups: 26% contained the motif W(TL/SV)K(L/X)PXW; 14% contained the motif AKTMP and 33% contained the motif KXPXW (Table 1).

**Table 1:** Amino acid sequence analysis of selected peptide-phages screened against D29 Fab-IgG.

Clones selected	Sequence	Cluster
P1	***W***SV***K***L***P***V***W***PINH	1
	GWPG**K**L**P**L**W**NWD	1
P2	Y**K**Q**P**L**W**PNWNKL	1
P3	Y**K**Q**P**L**W**PNQISW	1
P4	KPADVGELGKLY	1
	YKLPPLGWQWDG	1
	MPPMKPPLWPLE	1
P8	**AKTMP**WDLLFLL	2
	**AKTMP**GTYYSYW	2
P5	***W***TL***K***L***P***Q***W***LNST	2
	***W***TL***K***M***P***W***W***SSSL	2
	***W***SV***K***L***P***V***W***INHG	2
P9	KEPPDLAWLTRW	2
	GSSKVPMWLEW	2
P6	NHQH**K**I**P**L**W**NSW	3
P7	**K**L**P**M**W**ENWANYT	3
	TMKQPPRQWHFW	/
	SSTTYHSVISG	/
	QKLPPMGHLLFG	/

**Figure 4:**
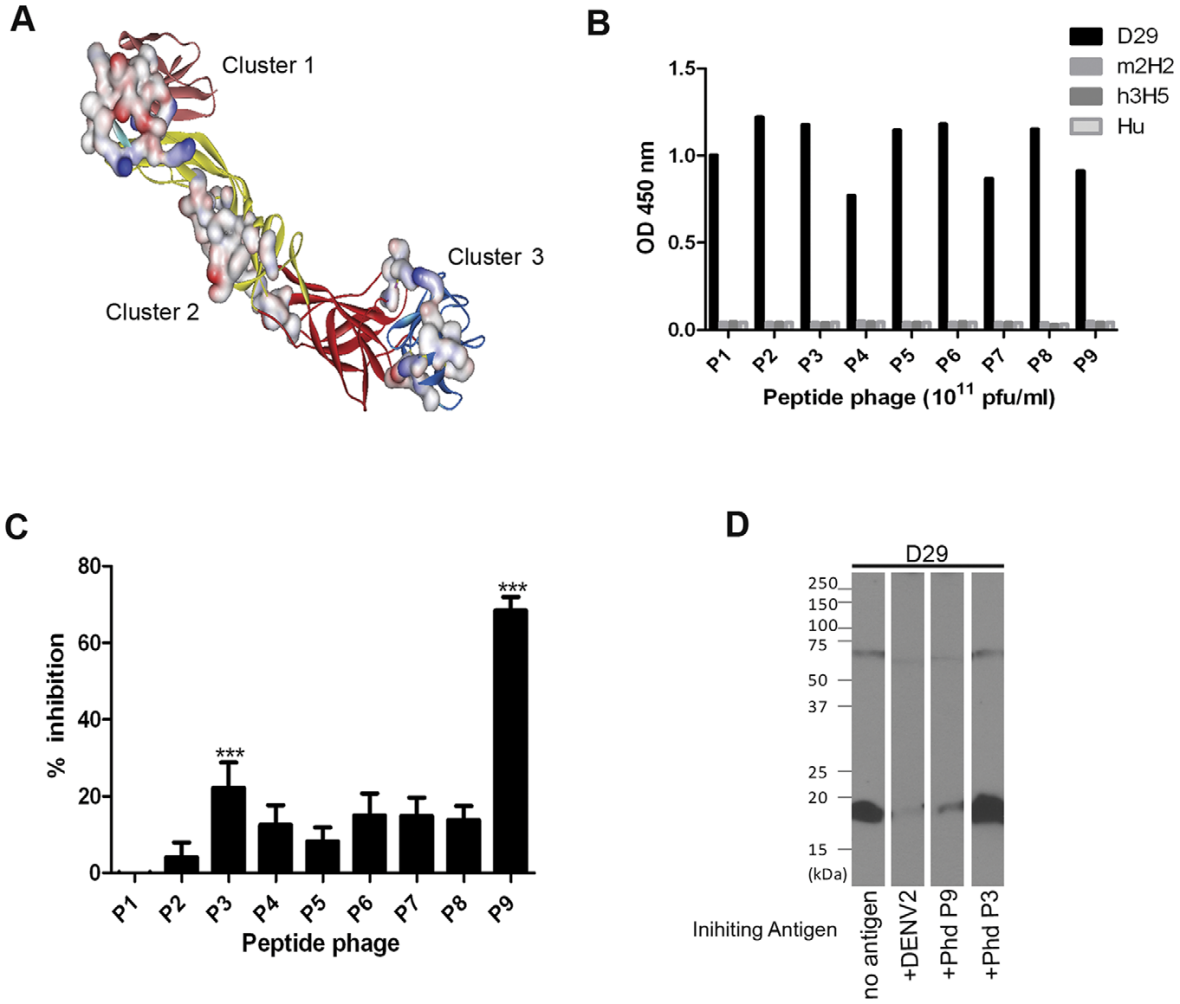
Localization of D29 Fab-IgG predicted epitopes in 3D crystal structures of DENV2 prM-E heterodimer (PDB 3C6E) and the binding specificity of peptide-phages. (A) Clusters of conformational epitopes predicted by Pepitope server are displayed on the prM-E heterodimer crystal structure at neutral pH with their solvent-accessible surfaces highlighted. The prM is pink, EDI is red, EDII is yellow, EDIII is blue, FP is cyan. (B) The binding specificity of peptide-phages (P1-P9) to D29 Fab-IgG was tested in a direct ELISA format with 10 µg/ml of D29 Fab-IgG, h3H5, m2H2 and non-DENV specific human antibody (Hu) immobilized on a Maxisorb plate. Ability of peptide-phages to inhibit D29 Fab-IgG binding to DENV2 was investigated by (C) ELISA and (D) Western blot analysis. For ELISA, peptide-phages (10^12^ pfu/ml) were incubated with D29 Fab-IgG for 1 hr at RT before application to immobilized DENV2 for 5 min at RT. Bound D29 Fab-IgG was detected with HRP-conjugated anti-Human IgG-Fc. The percentage of inhibition of D29 Fab-IgG binding by the peptide-phages shown is the average of three experiments. Error bars represent the standard errors of the mean (***p-value <0.005). For Western blot analysis, 0.5 µg/ml of D29 Fab-IgG was incubated with 8×10^6^ pfu of purified DENV2, 5% SM or 4×10^11^ pfu of peptide-phage clones for 1 hr at RT before applying to membrane transblotted with DENV2 viral lysate for 30 min at RT.

Mapping of peptide sequences using the Peptiope server against the 3D crystal structure of the prM-E heterodimer at neutral pH (PDB 3C6E) identified three main clusters of predicted epitope location. Since Pepitope assumes all input peptides mimic surface residues; all buried residues are eliminated from the search. The peptides with the KXPXW motif could mostly be mapped to the highest scoring Cluster 1 located at the prM/EDII interface. A few exceptions mapped to Cluster 3, most probably due to poorer sequence match and a lower alignment score. The remaining two motifs were mapped to the second highest scoring Cluster 2 that spans EDI and EDII (Fig. 4A).

**Figure 5:**
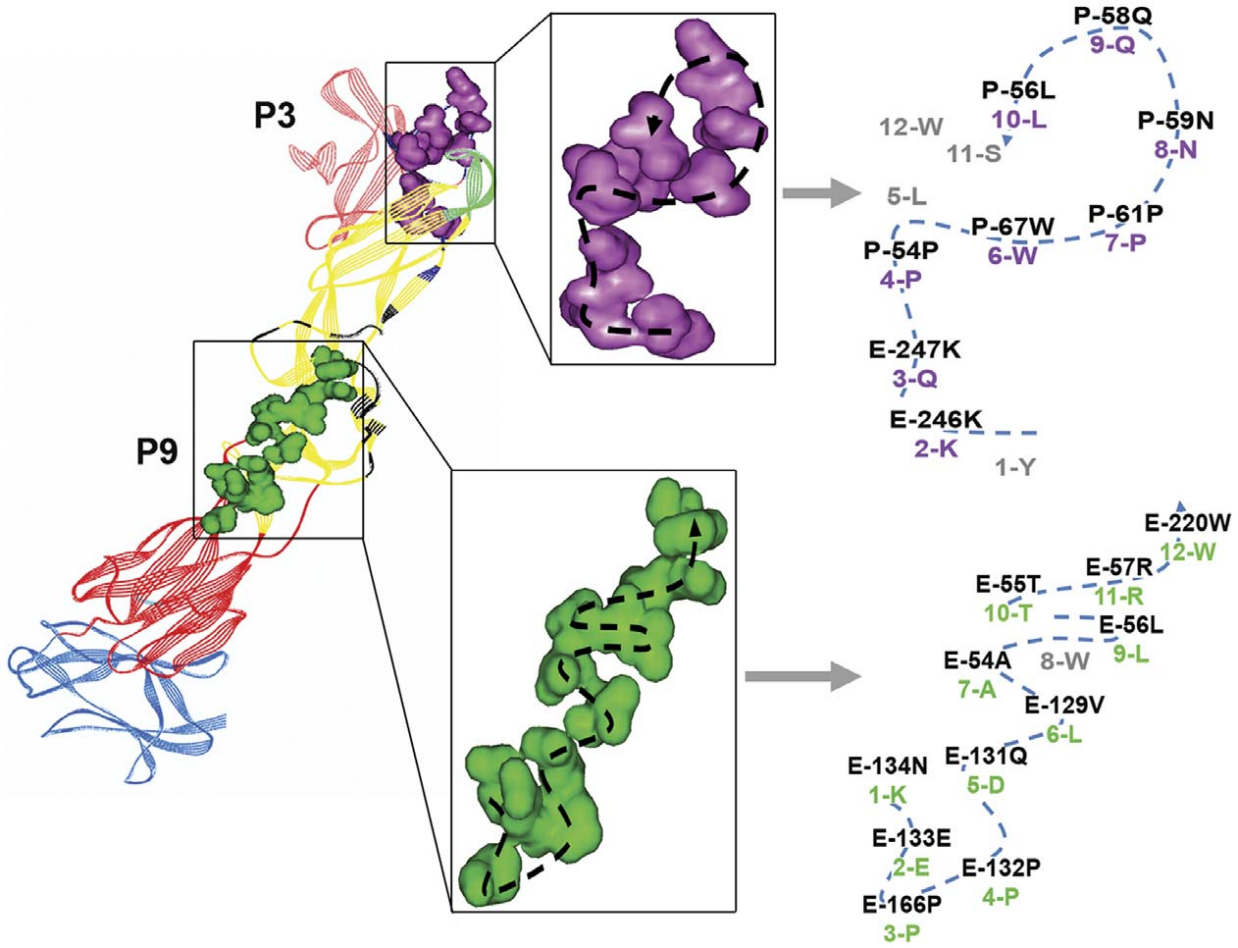
Localization of P3 and P9 peptide sequence on 3D crystal structure of prM-E heterodimer (PDB 3C6E). P3 (Purple) and P9 (green) peptide sequences were aligned with the predicted clusters (Cluster 1 – Navy blue; Cluster 2 – Black) on the prM-E crystal structure. The path of peptide phage sequences was highlighted with the participating residues from the cluster and the peptide phage labeled. Matched residues were purple (P3) or green (P9) and numbered accordingly; mis-matched residues were grey. The respective proteins and domains were highlighted as above.

To verify the specificity of the identified motifs, nine representative clones were selected (Table 1) and assessed for their ability to bind D29 Fab-IgG by direct ELISA. No cross-reaction by these peptide-phage clones with control antibodies was observed, confirming that the binding to D29 Fab-IgG was specific (Fig. 4B). The peptide-phage clones were then tested for their ability to inhibit D29 Fab-IgG’s binding to immobilized DENV2. Although most of the selected peptide-phage clones only weakly inhibited D29 Fab-IgG’s binding to DENV2, clone P3 (Cluster 1) and P9 (Cluster 2) significantly competed by 30% and 70%, respectively (Fig. 4C). Significant inhibition of binding to both prM and E was also observed on Western blot for P9, however no noticeable inhibition was observed for P3 (Fig. 4D). Alignment of P3 and P9 peptide sequences to their respective clusters revealed 9 and 11 aa match, respectively (Fig. 5). This may explain the difference in the ability of the two peptide phage clones to inhibit binding of D29 Fab-IgG.

### Site-directed Mutagenesis

**Figure 6:**
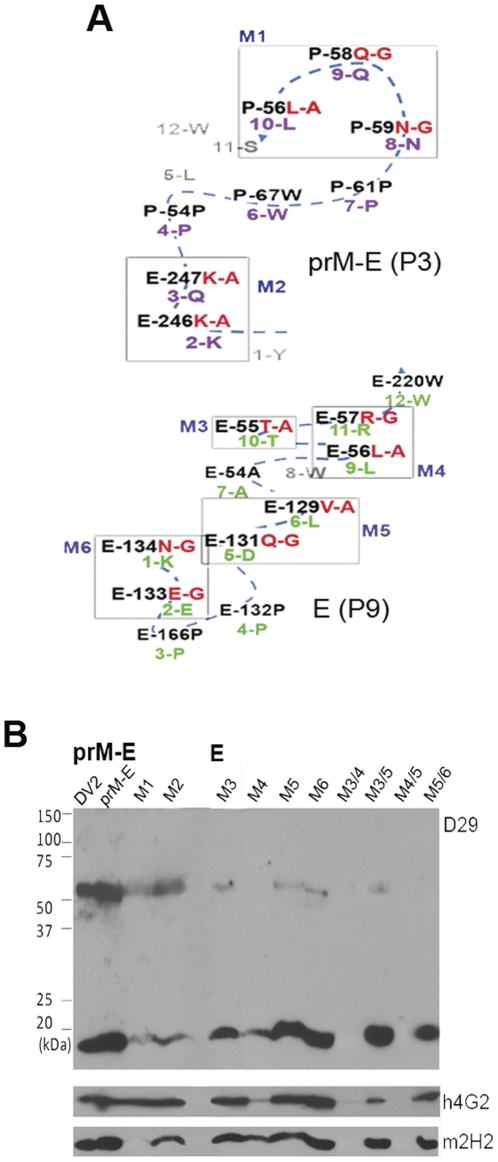
Site-directed mutagenesis of predicted epitope on prM-E protein. (A) To confirm the binding epitope of D29 Fab-IgG, residues within the P3 and P9 predicted sequences were mutated to generate mutants 1–6 (M1-6); M3/4, M3/5, M4/5 and M5/6 contain combinational-mutations as stated. (B) Reactivity of antibodies with the mutants was tested by Western blot analysis. Cleared lysate of DENV2-infected Vero cells (DV2), pCMV-prM-E- (prM-E) or mutants-transfected HEK 293 T cells (M1-5/6) were separated on 12% SDS-PAGE in non-reducing condition, followed by detection with h4G2, m2H2 and D29 Fab-IgG.

To confirm the residue assignation of the predicted epitopes for D29 on prM and E, clusters of 2–3 residue mutants were made on a prM-E construct that Puttikhunt and co-workers had previously shown to fold correctly when expressed in HEK293 [24] For the P3 (prM-E) epitope, two mutants, M1 (**P**56L-A, **P**58Q-G, **P**59N-G) and M2 (**E**246K-A, **E**247K-A) were generated (Fig. 6A). Their reactivity with D29 Fab-IgG was tested by Western blot analysis, using h4G2 and m2H2 as control antibodies. All antibodies reacted with their respective protein targets for the non-mutated prM-E protein; however, binding of m2H2 and D29 Fab-IgG to the prM protein was almost completely abolished by mutation at the prM residues in M1 (Fig. 6B). Mutation at the E residues within the P3 sequence (M2) on the other hand, did not have significant impact on the reactivity with D29 Fab-IgG (Fig. 6B). Detection of both M1 and M2 by h4G2 verified the expression of both mutants. To verify the P9 (E) epitope, eight mutants were generated: M3 (**E**55T-A), M4 (**E**56L-A, **E**57R-G), M5 (**E**129V-A, **E**131Q-G), M6 (**E**133E-G, **E**134N-G), M3/4 (**E**55T-A, **E**56L-A, **E**57R-G), M3/5 (**E**55T-A, **E**129V-A, **E**131Q-G), M4/5 (**E**56L-A, **E**57R-G, **E**129V-A, **E**131Q-G) and M5/6 (**E**129V-A, **E**131Q-G, **E**133E-G, **E**134N-G) (Fig. 6A). Both control antibodies were able detect their respective protein targets for all mutants, apart from M3/4 and M4/5 which appeared to be non-viable (Fig. 6B). The binding of h4G2 to E of M4 also appeared to be affected by the mutation introduced (Fig. 6B); likely to be caused by a change of local conformation, rather than a drop of expression level since prM-binding by m2H2 was normal. Notably, the binding of D29 Fab-IgG to the E protein of the M3, M5 and M6 mutants were severely affected; in particular, D29 Fab-IgG failed to detect E of M4 and M5/6, whereas binding of prM for all these mutants was not affected at all (Fig. 6B). Together these results confirm the epitopes identified through peptide-phage display.

### Antibody-dependent Neutralization and Enhancement

**Figure 7:**
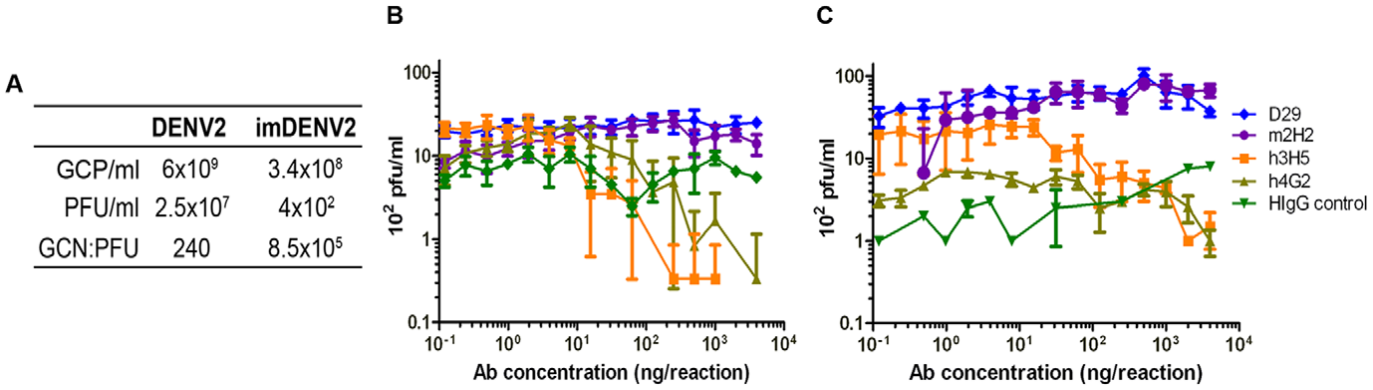
Effect of DENV maturation state on ADE profile by D29 Fab-IgG. (A) The specific infectivity of DENV2 and imDENV2 was determined. Serially diluted anti-DENV antibodies were incubated with (B) DENV2 or (C) imDENV2 at MOG 25 for 1 hr at 37^o^C before infection of 2×10^4^ K562 cells for 2 days. Viral titer was quantified by plaque assay (limit of detection is 0.5×10^2^ pfu/ml). Data presented is the average of three independent experiments with error bars representing the standard deviation.

Previous studies with anti-prM antibodies have shown that while the antibodies are generally non-neutralizing [9], [25], they are capable of rendering non-infectious immature DENV (imDENV) particles infectious through an FcγR-mediated process [9], [12]. Indeed, D29 Fab-IgG failed to neutralize any of the 4 serotypes of DENV at the highest concentration tested (400 µg/ml) in a PRNT assay (Table S1). Next, the ability of D29 Fab-IgG to enhance infection of DENV2 and imDENV2 was determined by carrying out ADE assays with FcγR-bearing K562 cells. The specific infectivity of the viruses was established by qPCR and plaque assay to be 8.5×10^5^:1 for imDENV2, which was significantly higher than that of the standard-grown DENV2 at 240:1 (Fig. 7A).

Enhancement of DENV2 infection was observed for all antibodies, with the exception of the non-DENV specific antibody. All the DENV-specific antibodies caused an approximately 20-fold increase in infection compared with IgG control (Fig 7B). As D29 Fab-IgG is non-neutralizing, it was capable of enhancing infection of prM-containing virus in the preparation for the entire range of concentrations tested.

Infection of virtually non-infectious imDENV was also significantly enhanced by D29 Fab-IgG to a level similar to m2H2, causing an 80–100 fold increase in infection relative to control IgG (Fig. 7C). The enhancement of imDENV infection by h3H5 was similar to that observed for DENV2, since its epitope was not affected by the maturity state hence it enhanced both equally well; h4G2 failed to enhance imDENV2 infection in K562 cells possibly be due to the occlusion of its epitope by the pr peptide [29], [30], and indicated by the immunoprecipitation protein profile (Fig. 3B).

## Discussion

To gain a deeper understanding of the early immune response against DENV, we have isolated and characterised a highly cross-reactive antibody fragment – D29, from a non-immune human Fab-phage library that shows near germline sequence. Upon conversion into full-length IgG, D29 showed cross-reactivity against all four serotypes of DENV and could be competed with the prM-specific mouse antibody 2H2. Intriguingly, D29 Fab-IgG was found to recognize both E and prM during Western blot analysis in a conformational dependant manner. Cross-reactivity between E and prM has been observed in antibodies isolated from mice as well as human patients [10], [11], [21]. However, the binding epitopes of these antibodies remain unknown and the observation is usually explained by general cross-reactivity of anti-prM antibodies.

Initial immunoprecipitation experiments with D29 Fab-IgG precipitated both prM and E proteins. However, upon dissociation of the prM interaction with E by the addition of SDS, only prM was precipitated by D29 Fab-IgG, pinpointing prM as the native binding target. Similarly, h3H5 only precipitated E upon addition of SDS, whereas the protein profile precipitated by h4G2 was not affected possibly due to its epitope at the fusion loop of E being occluded by the presence of prM [29], [30], and only ‘free’ E proteins were pulled down in both the presence and absence of SDS.

The epitope of D29 was mapped using a random-peptide phage display library, an approach which has been widely used for the identification of both linear and conformational epitopes [26], [31]–[34]. Two peptide phage clones, P3 (Cluster 1) and P9 (Cluster 2), displayed significant inhibition of D29 Fab-IgG binding to immobilized DENV2 by ELISA; P9 was also able to significantly block the binding of D29 Fab-IgG in a non-reducing Western blot analysis. This may be due to a better sequence match of P9 to its predicted cluster compared with P3 (Fig. 5), hence, a higher affinity of the peptide to D29 Fab-IgG. To ascertain the authenticity of the predicted epitopes, the predicted residues were confirmed by site-directed mutagenesis of the P3 and P9 peptide sequence. The residues **P**56L, **P**58Q and **P**59N within the P3 epitope were found to be the principle residues involved in the interaction between prM and D29 Fab-IgG; they were also critical for the binding of m2H2, corroborating with results of competition assays. The binding for E protein was not really affected by mutation of aa residues within the P3 epitope; contrasting to the significant impact caused by mutation of 1–2 residues within the P9 epitope. The combined mutation of **E**129V, **E**131Q, **E**133E, and **E**134N completely abolished binding of D29 Fab-IgG to E protein, establishing the critical residues within the P9 epiotpe. Taken together, these results suggest that D29 Fab-IgG recognises two epitopes on DENV – a solvent-accessible epitope on prM as the principle binding epitope, and a cryptic epitope on E which mimics the prM epitope but is not available on a native, functionally-folded E protein. The P3 and P9 epitopes are highly conserved across the four DENV serotypes, as well as two other flaviviruses, Japanese encephalitis virus and West nile virus; but with increasing divergence in Yellow fever virus and Tick-borne encephalitis virus (Fig. S4).

Most current dengue vaccines consist of both prM and E proteins [12], [13], [35]; studies performed with tick-borne encephalitis virus indicate that proper folding of E requires the chaperone function of prM [36]. However, as demonstrated in this study and recent reports, prM-specific antibodies are able to restore and enhance the infectivity of imDENV and partially mature DENV [9], [11], [12]; it may be worthwhile to consider alternative vaccine approaches that minimize anti-prM responses during vaccine design [9]. The findings from our study also suggest that any partial denaturation or unfolding of an E protein vaccine preparation may result in the exposure of a cryptic P9-like epitope, which has the potential to induce D29-like antibodies with threatening ADE capability.

A recent study investigating the acute and early convalescent B cell response in dengue patients has found dengue virus to have a significant B cell activation capacity, causing a transient but high appearance of plasmablast and plasma cell, coinciding with that of dengue-specific IgG antibodies at day 4–7 after onset of fever [37]. The similarity of D29 Fab-IgG to germline sequence suggests that such antibodies are present in the immune repertoire of naïve individuals and given the inherent high affinity of D29, such antibodies may be preferentially selected during clonal expansion and maintained as memory. Indeed, antibodies that cross-react between prM and E has been isolated from memory B cells in primary and secondary dengue patients [9], [11]. The original immunogens for these cross-reactive antibodies are difficult to determine since they were generated in the course of natural infections, but it would be interesting to identify the epitope and germline of these antibodies and compare the sequences with D29 Fab-IgG.

## Supporting Information

Figure S1 **Binding enrichment of Fab phage and SDS-PAGE analysis of D29 Fab-IgG.** (A) Purified DENV2 was coated on Maxisorb plate and polyclonal phage after each round of panning was added at 1∶10 v/v dilution along with unpanned phage as control. Bound phages were detected with HRP-conjugated anti-M13 monoclonal antibody. (B) D29 was converted into human IgG format and expressed in HEK 293 T cells. The quality of resulting IgG was analysed by resolving 2.5 µg of antibody on 12% SDS-PAGE with (lane 1) or without (lane 2) the presence of DTT.
(TIF)

Figure S2 **Alignment of D29 Fab-IgG with germline sequence.** The amino acid sequence of D29 IgG was aligned with the germline sequence using the on-line program IgBlast. CDR - (complementarity determining region); HC – antibody heavy chain; LC - antibody light chain.
(TIF)

Figure S3 **Epitope mapping using synthetic linear peptides.** In attempt to identify D29 Fab-IgG’s target on E and prM, 10 µg/ml of synthetic peptides corresponding to prM (A) and E (B) was incubated with D29 Fab-IgG. Peptides corresponding to E were tested individually but presented as groups of 5. For all experiments, 2×10^6^ pfu/ml of DENV2 or imDENV2 was included as control antigen. M1-32: 15 mer peptides corresponding to prM. Pr1-5: >20 mer custom-made peptides covering parts of prM. E1-73: 15 mer peptides corresponding to E. (**p-value<0.005)
(TIF)

Figure S4 **Alignment of residues corresponding to the D29 epitopes.** Residues of flaviviruses (from top to bottom – DENV1 Hawaii, DENV2 16881, DENV3 H87, DENV4 H241, Japanese encephalitis virus (JEV) P3, West Nile virus (WNV) NY2003suffolk, Yellow fever virus (YV) 17D204USA, Tick-borne encephalitis virus (TBEV) western subtype vaccine strain Neudoerfl) corresponding to P3 and P9 epitopes are aligned. Residues identical to the predicted sequence are depicted as dots.
(TIF)

Materials and Methods S1 Click here for additional data file. (DOC)

Table S1 **PRNT_50_ value of DENV-specific antibodies.**
(DOCX)
