# Supplementary figures and images for: A Human PrM Antibody That Recognizes a Novel Cryptic Epitope on Dengue E Glycoprotein
Source: PLoS One. 2012 Apr 3;7(4):e33451. doi: 10.1371/journal.pone.0033451 (PMC3317930; doi:10.1371/journal.pone.0033451)

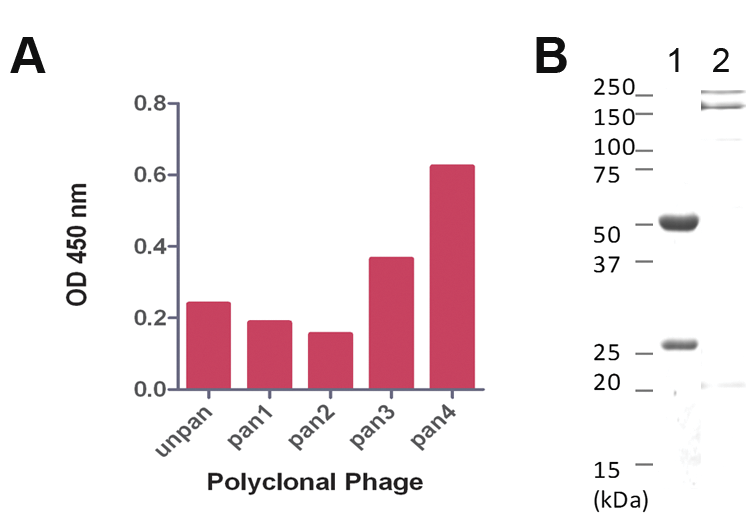

Supplement: Figure S1 — Binding enrichment of Fab phage and SDS-PAGE analysis of D29 Fab-IgG. (A) Purified DENV2 was coated on Maxisorb plate and polyclonal phage after each round of panning was added at 1∶10 v/v dilution along with unpanned phage as control. Bound phages were detected with HRP-conjugated anti-M13 monoclonal antibody. (B) D29 was converted into human IgG format and expressed in HEK 293 T cells. The quality of resulting IgG was analysed by resolving 2.5 µg of antibody on 12% SDS-PAGE with (lane 1) or without (lane 2) the presence of DTT. (TIF) [file pone.0033451.s001.tif]

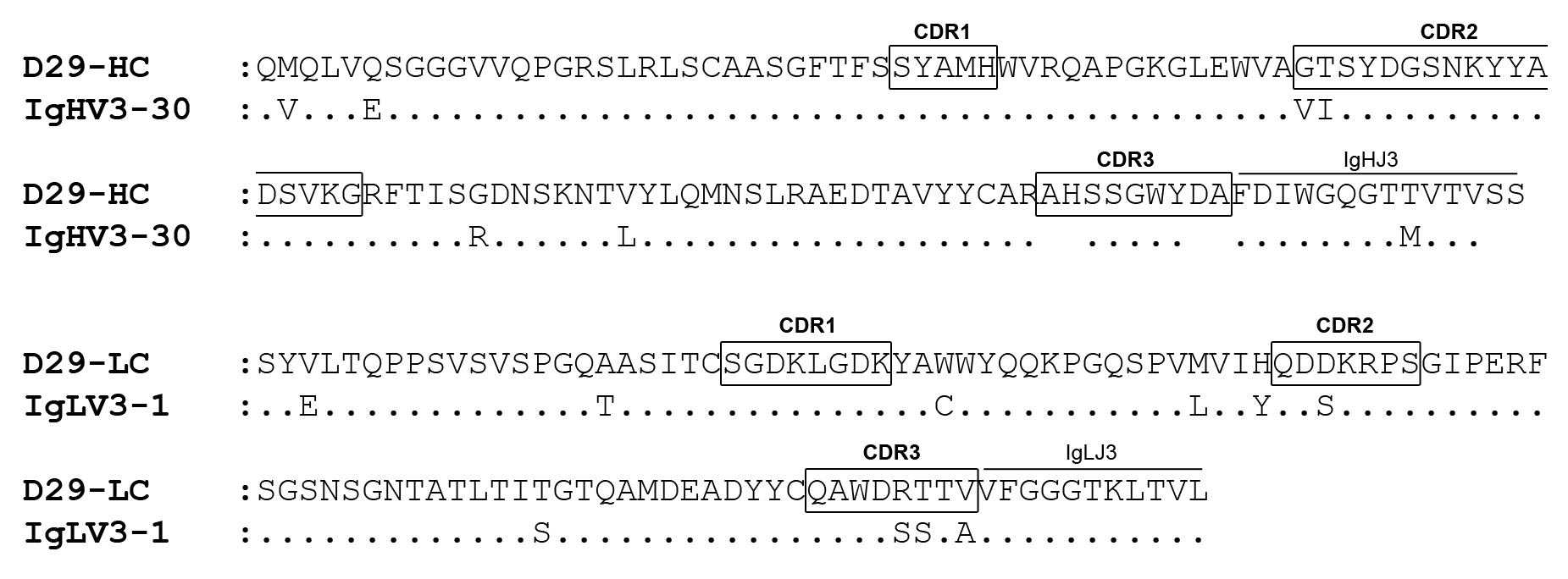

Supplement: Figure S2 — Alignment of D29 Fab-IgG with germline sequence. The amino acid sequence of D29 IgG was aligned with the germline sequence using the on-line program IgBlast. CDR - (complementarity determining region); HC – antibody heavy chain; LC - antibody light chain. (TIF) [file pone.0033451.s002.tif]

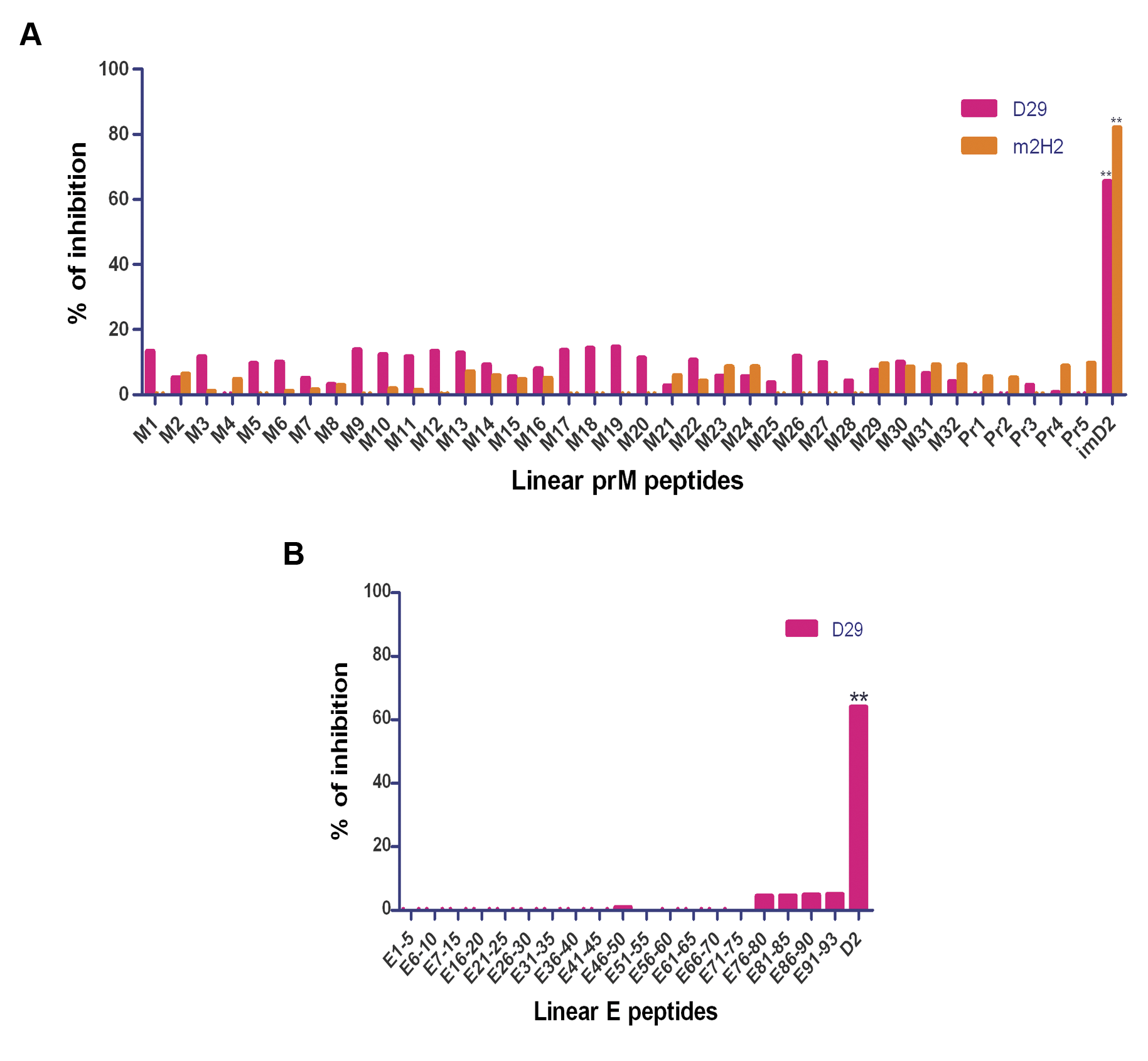

Supplement: Figure S3 — Epitope mapping using synthetic linear peptides. In attempt to identify D29 Fab-IgG’s target on E and prM, 10 µg/ml of synthetic peptides corresponding to prM (A) and E (B) was incubated with D29 Fab-IgG. Peptides corresponding to E were tested individually but presented as groups of 5. For all experiments, 2×10^6^ pfu/ml of DENV2 or imDENV2 was included as control antigen. M1-32: 15 mer peptides corresponding to prM. Pr1-5: >20 mer custom-made peptides covering parts of prM. E1-73: 15 mer peptides corresponding to E. (**p-value<0.005) (TIF) [file pone.0033451.s003.tif]

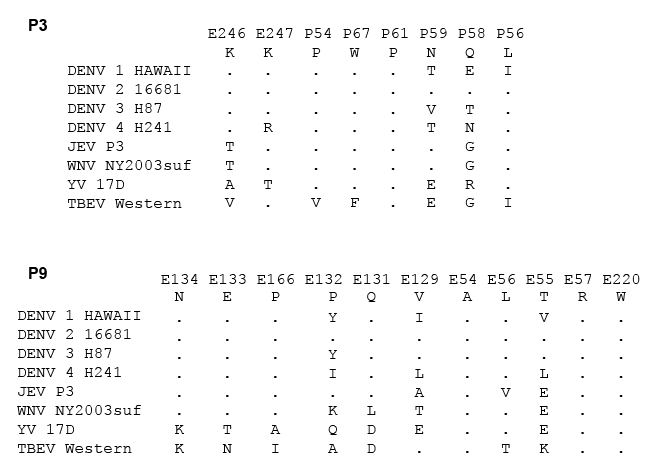

Supplement: Figure S4 — Alignment of residues corresponding to the D29 epitopes. Residues of flaviviruses (from top to bottom – DENV1 Hawaii, DENV2 16881, DENV3 H87, DENV4 H241, Japanese encephalitis virus (JEV) P3, West Nile virus (WNV) NY2003suffolk, Yellow fever virus (YV) 17D204USA, Tick-borne encephalitis virus (TBEV) western subtype vaccine strain Neudoerfl) corresponding to P3 and P9 epitopes are aligned. Residues identical to the predicted sequence are depicted as dots. (TIF) [file pone.0033451.s004.tif]
