# Supplementary material for: A Human PrM Antibody That Recognizes a Novel Cryptic Epitope on Dengue E Glycoprotein
Source: PLoS One. 2012 Apr 3;7(4):e33451. doi: 10.1371/journal.pone.0033451 (PMC3317930; doi:10.1371/journal.pone.0033451)
Supplement: Materials and Methods S1 [file pone.0033451.s005.doc]

**Supporting information**

**Methods and Materials S1**

**Panning for human Fab from a phage display library**

The four serotypes of purified Dengue virus were screened sequentially according to their E protein amino acid sequence relatedness: 4-2-3-1 using a non-immunized human Fab phage display library HX01 (Humanyx Pte Ltd, Singapore). The screening procedures were as described previously [15] except for the elution of phage by trypsin cleavage (500μL of 2mg/ml trypsin solution) for 30min at 37°C.

**Selection of unique Fab clones and conversion into Human IgG1**

To select for individual antigen-binding Fab clones, single colonies were picked as described previously [15] and screened against DENV2 using standard ELISA method. BstN1 fingerprinting of the PCR-amplified Fab coding regions was carried out to assess the uniqueness of positive clones. The identity of unique clones were verified by sequencing using primers specific for the regions of the vector flanking the variable heavy and variable light chains of the antibody fragment.

The distinct immunoglobulin sequences were converted into human full length IgG1 by cloning the Fab into pCMV-Fab-IgG1 vector, expressed in HEK293T cells and purified from the culture supernatant using recombinant Protein A sepharose (Pierce), as previously described [15]. Purity and integrity of the Fab-IgGs was verified using SDS-PAGE analysis with or without the presence of dithiothreitol (DTT) (Unpublished data).

**Peptide inhibition ELISA**

To elucidate the epitope of D29 Fab-IgG, 5 long (>20 amino acids) linear peptides spanning the length of prM of DENV2 were adapted from a study by Vazquez *et al*. [24] and a library of 15-mer synthetic linear peptides overlapping by 10 amino acid (aa) (Mimotope, Clayton) (a kind gift from Dr. Antonio Bertolleti, Infection and Immunity Programme, Singapore Institute of Clinical Sciences) corresponding to aa1-114 of prM and aa1-166 of E was tested for its ability to inhibit the binding of Fab-IgG D29 to its natural target epitope using the method described previously [18]. Antibodies at the optimal sub-saturation level (previously determined by serial titration against DENV2 with the same length of incubation time as the inhibition ELISA) were incubated with 5µg of each peptide or 2x10^6^pfu/well of DENV2 for 1hr at RT before application to the a plate coated with DENV2 for 5min at RT followed by 4 PBST washes. Bound antibody was detected with HRP-conjugated anti-human IgG-Fc or anti-mouse IgG Fc secondary antibody for 1hr at RT and developed as described above.
