# Supplementary material for: A Human PrM Antibody That Recognizes a Novel Cryptic Epitope on Dengue E Glycoprotein
Source: PLoS One. 2012 Apr 3;7(4):e33451. doi: 10.1371/journal.pone.0033451 (PMC3317930; doi:10.1371/journal.pone.0033451)
Supplement: Table S1 — PRNT50 value of DENV-specific antibodies. (DOCX) [file pone.0033451.s006.docx]

Table S1

| PRNT_50_ value of DENV-specific antibodies. | | | | |
| --- | --- | --- | --- | --- |
|  | **PRNT_50_(μg)^a^** | | | |
|  | DENV1 | DENV2 | DENV3 | DENV4 |
| m3H5 | - ^b^ | 0.125 | - | - |
| m4G2 | - | 1 | - | 1 |
| D29 | - | - | - | - |

^a^50% plaque reduction neutralization end-points titer (PRNT) in Vero cells calculated by probit analysis.

^b^ No neutralization observed with up to 4μg of antibody
